# Primary extranodal marginal zone B-cell lymphoma with diffuse uveal involvement and focal infiltration of the trabecular meshwork: a case report and review of literature

**DOI:** 10.1186/s12886-015-0038-7

**Published:** 2015-05-07

**Authors:** Xinxiao Gao, Bin Li, Qisheng You, Xiaoyan Peng

**Affiliations:** Beijing Institute of Ophthalmology, Beijing Ophthalmology and Visual Science Key Lab, Beijing Tongren Eye Center, Beijing Tongren Hospital, Capital Medical University, 17 Hougou Lane, Chongnei Street, Beijing, 100005 China; Department of Ophthalmology, Beijing Anzhen Hospital, Capital Medical University, Beijing, China

## Abstract

**Background:**

Primary extranodal marginal zone lymphoma (EMZL) of the uvea is a rare condition and diagnosis may be challenging. We aim to report the clinical, histopathologic and immunohistochemical findings in a case of primary EMZL with diffuse uveal involvement and focal infiltration of the trabecular meshwork.

**Case presentation:**

A 38-year-old male presented with 2-year progressive vision loss in the right eye. Fundus examination showed choroidal thickening with diffuse retinal pigment epithelium (RPE) changes and inferior exudative retinal detachment. Ultrasonography revealed low-reflective masses with diffuse thickening of the choroid involving the optic nerve and orbit. Despite treatment with steroids, his symptoms progressed over time. One year later, visual acuity of the right eye markedly decreased to no light perception and enucleation was performed. Histopathological findings revealed infiltrates of malignant cells in the choroid, iris, ciliary body and trabecular meshwork. Immunohistochemistry confirmed the diagnosis of primary uveal EMZL.

**Conclusions:**

This is the first case reporting primary EMZL diffusely involving the uvea with focal infiltration of the trabecular meshwork.

## Background

Ophthalmic lymphomas account for approximately 10% of all extranodal malignant lymphomas [1,2]. The major subtype of ophthalmic lymphoma is extranodal marginal zone lymphoma (EMZL) of mucosa-associated lymphoid tissue (MALT) [3,4]. It presents an indolent clinical course and usually involves the conjunctiva, lacrimal gland, and orbit. Intraocular choroidal involvement is a rare condition. Primary iris and ciliary body EMZL are exceptionally rare with only a handful of cases reported in the literature [5-7].

In this study, we present a rare case of EMZL diffusely involving the uvea with focal involvement of trabecular meshwork and review of reported cases. The study was approved by institutional review committee of Beijing Tongren Hospital, Capital Medical University and was conducted in accordance with the tenets of the Declaration of Helsinki. Written informed consent was obtained from the patient.

## Case presentation

**Figure 1:**
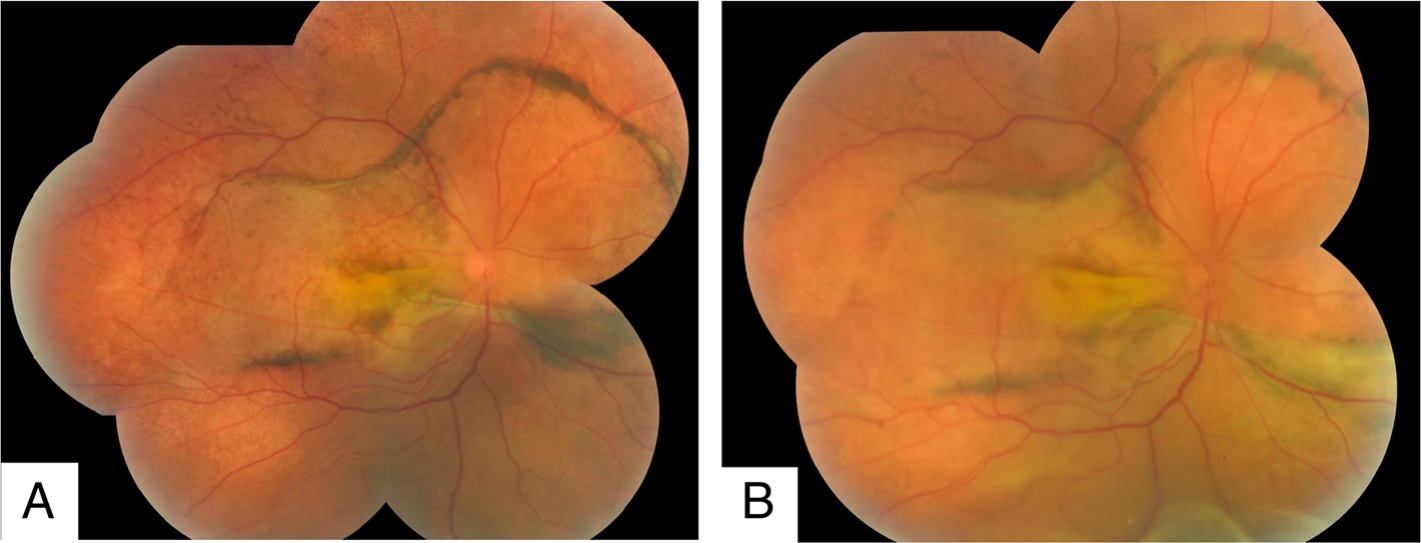
Fundus colour photograph. **(A)** Choroidal thickening was shown, with diffuse retinal pigment epithelium (RPE) changes and inferior exudative retinal detachment. **(B)** Deteriorated retinal detachment was demonstrated.

**Figure 2:**
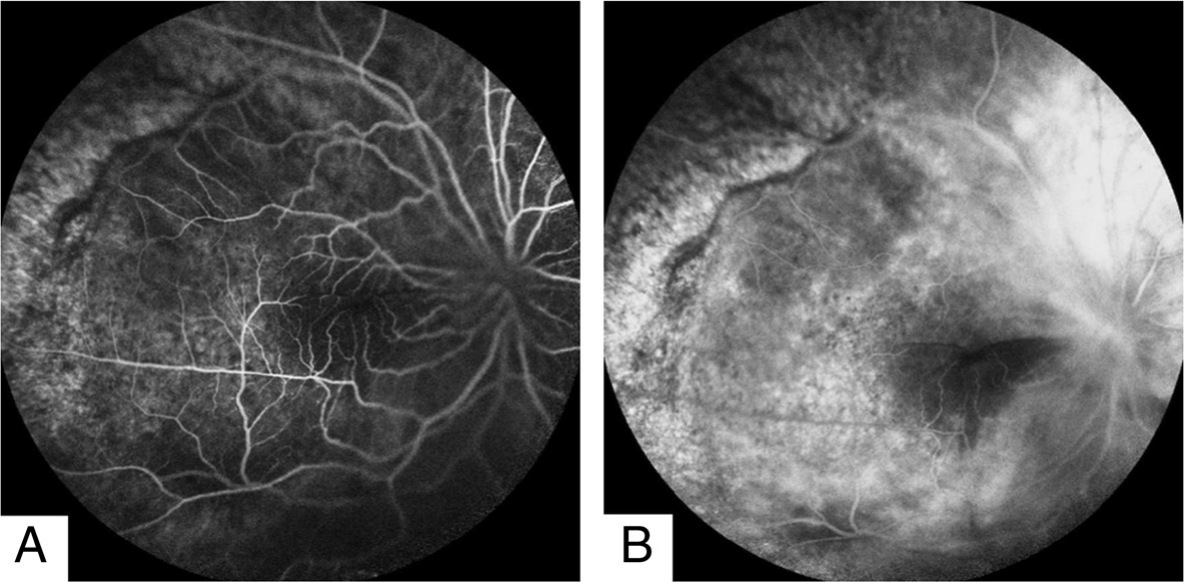
Fluorescence angiography. **(A)** Early-phase fluorescein angiogram of right eye. **(B)** Late phase showed macular edema and nonspecific leakage at the level of the RPE.

**Figure 3:**
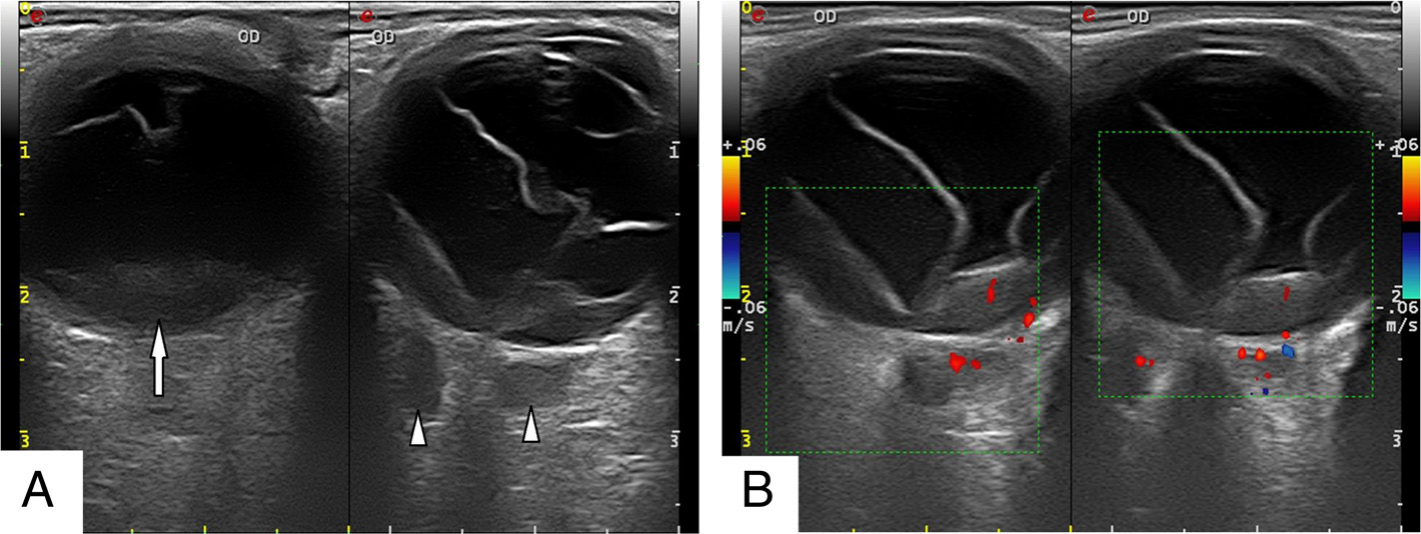
Ultrasonography. **(A)** B scan showed diffuse thickening of choroid (arrow) with low-reflective retrobulbar masses (arrow heads). **(B)** Colour Doppler imaging revealed choroidal infiltration and involvement of optic nerve and orbit.

In November 2012, a 38-year-old male presented with 2-year progressive vision loss in his right eye. He was referred to our clinic for a choroidal lesion with subretinal fluid in the right eye. He had been generally healthy and denied any significant past medical history including diabetes mellitus and hypertension. Since 2010, the onset of the vision decrease, a flat serous retinal detachment in his right eye was detected by a local ophthalmologist. However, no treatment was given, for the diagnosis was unclear at that moment. After several outpatient follow-ups, the disease progressed gradually. One year later, he visited another ophthalmologist for a second opinion. At that time, a choroidal mass with associated serous retinal detachment was detected, yet no treatment was performed, for the diagnosis remained unclear. At his initial visit to our institute, best corrected visual acuity was 20/200 in the right eye and 20/16 in the left eye. Intraocular pressure of both eyes was within normal limits. Examination of the left eye was unremarkable. Right eye anterior segment was normal. Fundus examination revealed choroidal thickening with diffuse retinal pigment epithelium (RPE) changes and inferior exudative retinal detachment (Figure 1A). Fluorescein angiography showed nonspecific leakage at the level of the RPE and formation of macular edema, combined with thickening of the choroid and serous retinal detachment (Figure 2A-B). Ultrasonography showed low-reflective masses with diffuse thickening of the choroid and involvement of optic nerve and orbit (Figure 3A-B). A suspicious diagnosis of posterior scleritis or choroidal hemangioma was made. Retrobulbar twenty-milligram triamcinolone acetonide was then given. However, the ocular findings were unchanged. One week later, he received experimental treatment of intravitreal ranibizumab.

**Figure 4:**
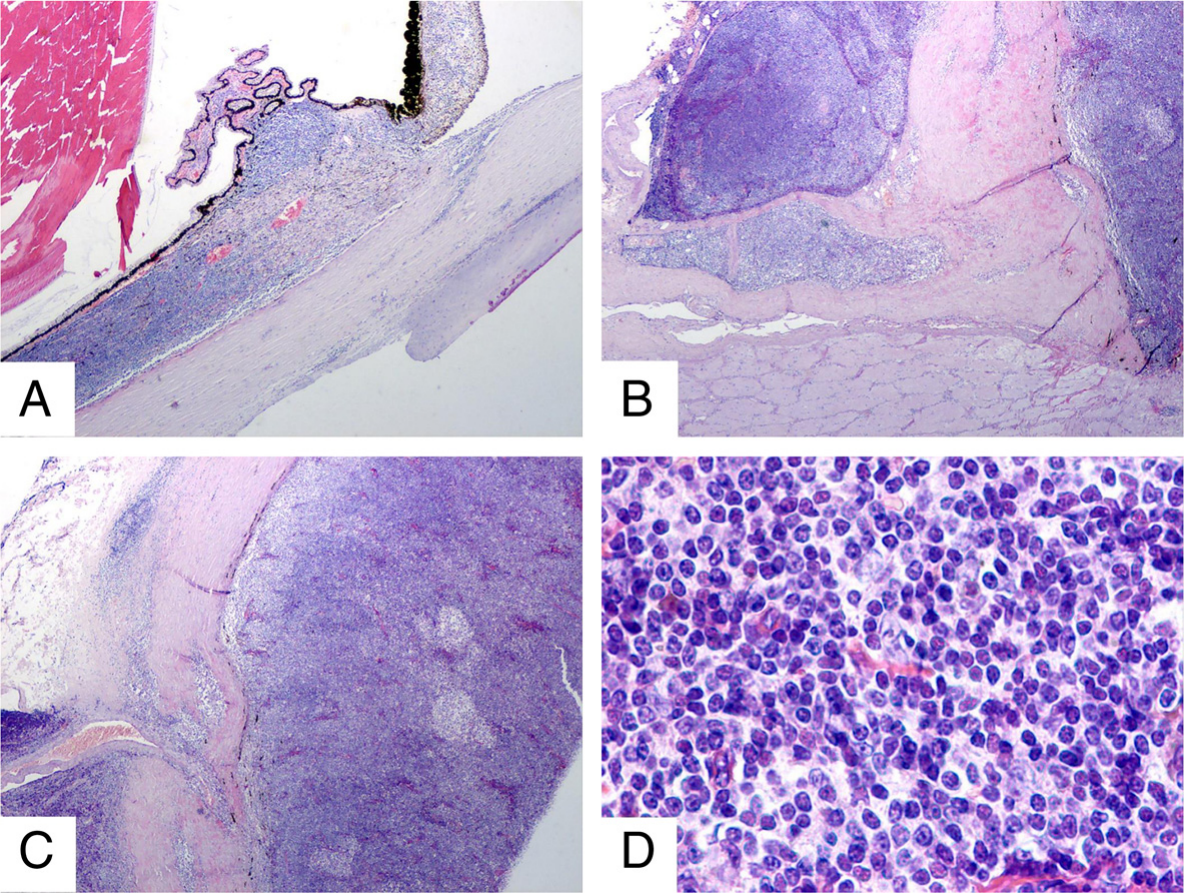
Histologic features of EMZL. **(A)** Low power magnification showing extensive infiltration of uvea, including choroid, iris, ciliary body and trabecular meshwork (HE; magnification × 20). **(B)** Higher power magnification demonstrating choroidal infiltration by tumor cells, with involvement of optic nerve and orbit (HE; magnification × 40). **(C)** Increased magnification illustrating the infiltrate of tumor cells with heavily pigmented lymphocytes in choroid and extension of malignant cells to retrobulbar tissue through sclera emissary canal (HE; magnification × 40). **(D)** High power magnification revealing an infiltrate of atypical lymphocytes with small irregular nuclei (HE; magnification × 400).

**Figure 5:**
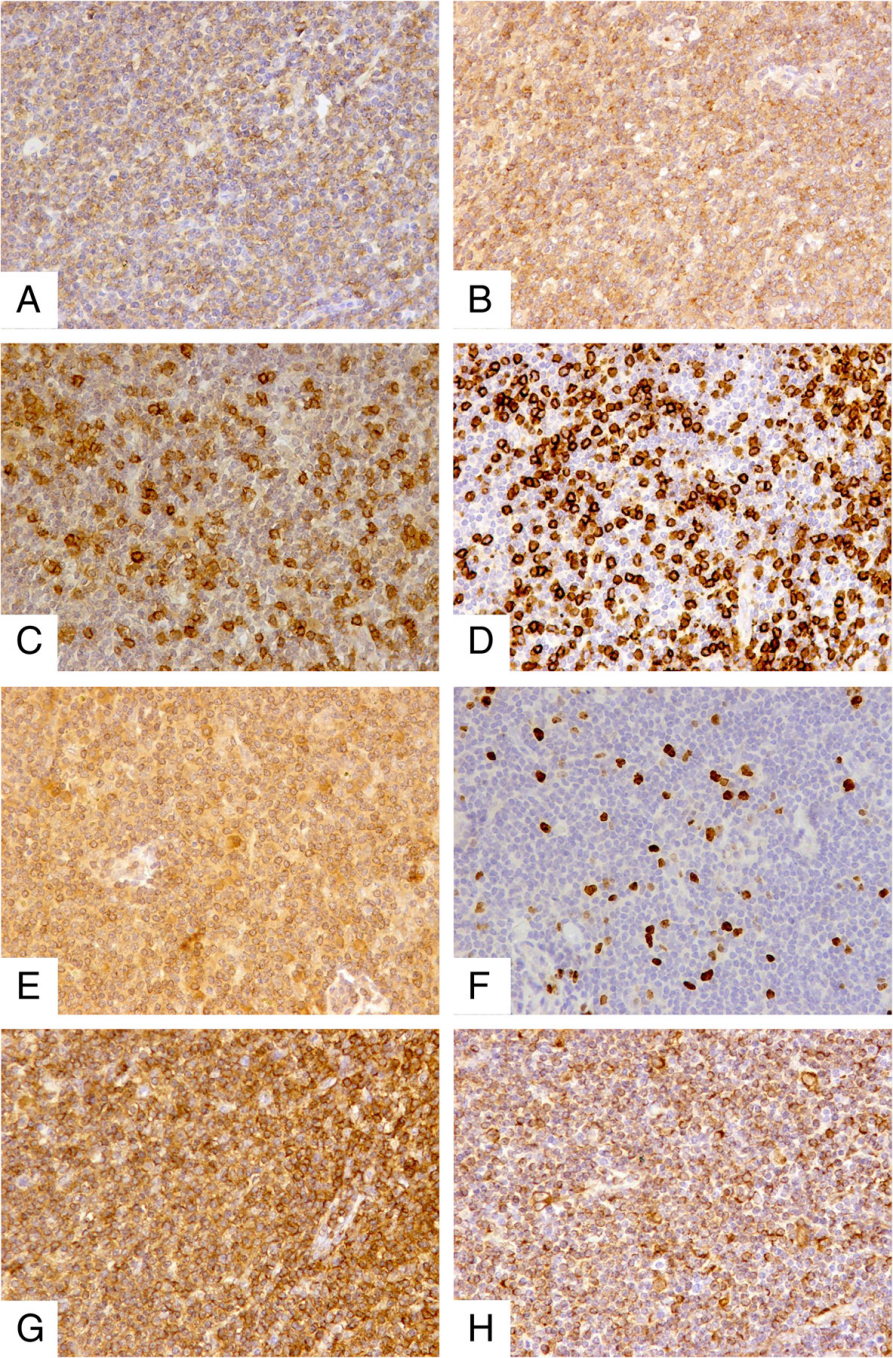
Immunohistochemical expression of EMZL. **(A)** Tumour cells positive for CD20. **(B)** Positivity for CD79a. **(C)** Positivity for CD5. **(D)** Positivity for CD45RO. **(E)** Positivity for Bcl-2. **(F)** Positivity for Ki67. **(G)** Positivity for LCA. **(H)** Positivity for Vimentin.

The patient was loss to follow up until 6 months after intravitreal ranibizumab injection. Magnetic resonance imaging (MRI) of the orbits confirmed extensive choroidal thickening involving the optic disc with a height of 0.37 cm. Two lobulated soft tissue masses were seen around the optic nerve. Fundoscopy revealed deteriorated retinal detachment (Figure 1B). The patient and his family refused to undergo aggressive treatment, including biopsy. One year later, patient returned to our clinic with visual acuity of no light perception on the right eye. Since the etiology of the mass was uncertain, enucleation was performed after written informed consent was obtained from the patient. Histopathology revealed the choroid, iris, ciliary body and trabecular meshwork infiltrated with malignant cells (Figure 4A). Orbit and optic nerve were also involved (Figure 4B). Intra-retinal exudates were noted and the retina was detached with subretinal exudation and some foamy cells. Fibrous membrane was noted between the retina and a diffusely thickened choroid. This was found to be heavily pigmented lymphocytes with small irregular nuclei. Extension of malignant cells to the retrobulbar tissue was also noted (Figure 4C-D). Immunohistochemistry showed positive staining for CD5, CD20, CD23, CD38, CD79a, CD45RO, Bcl-2, Kappa, Lambda, LCA, Vimentin and Ki-67 (5%-10% positive cells), whereas CD10, Bcl-6 and Cyclin D1 were negative (Figure 5A-H). The diagnosis of primary uveal EMZL was made based on the histomorphology and immunohistochemistry examinations. The patient underwent a systemic work up including positron emission tomography and bone marrow aspiration smears. The results were negative.

## Discussion

Primary uveal lymphoma has been named as “uveal lymphosarcoma”, “reactive lymphoid hyperplasia” and “uveal pseudotumours” in the past [8-10]. In 1994, based on the findings of the International Lymphoma Study Group, the term ‘extranodal marginal zone B cell lymphoma’ was proposed by the Revised European American Lymphoma (REAL) classification to incorporate both mucosa- and non-mucosa-associated ‘MALT’ lymphomas [11]. More recently, primary uveal lymphoma was suggested to be accurately subtyped as EMZL of MALT type (WHO lymphoma classification), since its morphological, immunophenotypical, and clinical features were identical to those of EMZL-MALT elsewhere [12].

According to the REAL classification, only 61 EMZL cases (including the present case) have been reported since 1994 (More details can be found in Table 1) [5-7,12-23]. These cases included 24 male and 13 female (patient gender was not reported in 24 cases). The age of the patients at presentation ranged from 33 to 85 years. Majority of the cases involved the choroid. Primary iris lymphomas were unusual, with only 2 cases described [5,6]. And only a single case of primary lymphomas limited to the ciliary body has been reported [7]. Most of these cases involved extraocular tissue, with only 10 cases having no extraocular extension [24].

**Table 1:** **Literature review of EMZL since 1994**

**No. of cases**	**Ref/Year**	**Age/Gender**	**Eye/Initial BCVA**	**Extraocular extension**	**Site**	**Pathology findings**	**Treatment**	**Systemic work-up**	**Follow-up (months)**
1	5/2000	51/F	L/0.5	N	Iris	CD20+, CD19+, Kappa LC+	Radiotherapy Enucleation	N	72
2	12/2002	63/M	NA/NLP	N	Uvea	CD79a+, CD20+, BSAP+, MUM1+, CD38+, CD138-, Vs38c+, Kappa LC-, Lambda LC+, IgM-, IgD-	Enucleation	N	156
3		40/F	NA/NLP	Y	Uvea	CD79a+, CD20+, BSAP+, MUM1+, CD38+, CD138-, Vs38c-, Kappa LC-, Lambda LC+, IgM+, IgD-	Enucleation, Radiotherapy	Y	300
4		40/M	NA/HM	Y	Uvea	CD79a+, CD20+, BSAP+, MUM1+, CD38+, CD138±, Vs38c+, Kappa LC+, Lambda LC-, IgM+, IgD-	Enucleation, Radiotherapy	N	216
5		45/M	NA/LP	Y	Uvea	CD79a+, CD20+, BSAP+, MUM1+, CD38+, CD138+, Vs38c+, Kappa LC-, Lambda LC+, IgM+, IgD-	Enucleation, Radiotherapy	N	120
6		64/M	NA/LP	Y	Uvea	CD79a+, CD20+, BSAP+, MUM1+, CD38-, CD138+, Vs38c+, Kappa LC-, Lambda LC+, IgM+, IgD-	Enucleation	N	108
7		33/M	NA/CF	Y	Uvea	CD79a+, CD20+, BSAP+, MUM1+, CD38+, CD138-, Vs38c+, Kappa LC-, Lambda LC+, IgM+, IgD-	Enucleation	N	84
8		63/M	NA/0.05	Y	Uvea	CD79a+, CD20+, BSAP+, MUM1+, CD38+, CD138-, Vs38c+, Kappa LC+, Lambda LC-, IgM+, IgD-	Enucleation	N	60
9		59/F	NA/LP	Y	Uvea	CD79a+, CD20+, BSAP+, MUM1+, CD38+, CD138-, Vs38c-, Kappa LC-, Lambda LC-, IgM+, IgD-	Enucleation	N	Unknown
10		66/M	NA/LP	Y	Uvea	CD79a+, CD20+, BSAP+, MUM1+, CD38+, CD138-, Vs38c-, Kappa LC-, Lambda LC+, IgM+, IgD-	Enucleation	N	Unknown
11		73/M	NA/LP	Y	Uvea	CD79a+, CD20+, BSAP+, MUM1+, CD38+, CD138+, Vs38c-, Kappa LC+, Lambda LC-, IgM+, IgD-	Enucleation	Y	2
12		81/M	NA/LP	Y	Uvea	CD79a+, CD20+, BSAP+, MUM1+, CD38+, CD138+, Vs38c-, Kappa LC-, Lambda LC+, IgM+, IgD-	Enucleation	Y	180
13		73/F	NA/NLP	Y	Uvea	CD79a+, CD20+, BSAP+, MUM1+, CD38+, CD138+, Vs38c+, Kappa LC+, Lambda LC-, IgM+, IgD-	Enucleation	N	Unknown
14		63/M	NA/N LP	N	Uvea	CD79a+, CD20+, BSAP+, MUM1+, CD38+, CD138+, Vs38c+, Kappa LC+, Lambda LC-, IgM+, IgD-	Enucleation	N	120
15	6/2003	83/F	L/0.2	N	Iris	CD20/l-26+, CD79a+, CD3-, CD45RO/UCHL-1-	Radiotherapy	N	8
16	13/2005	45/M	L/0.16	N	Choroid	CD79a+, CD20+, BCL2+, CD43+, CD3-, CD5-, CD23-, CyclinD1-, Kappa LC+, IgM+, Ki67 10%	Radiotherapy	N	17
17	7/2008	84/F	R/0.63	N	Ciliary body	CD79a+, CD20+, CD43±, IgM+, CD5-, CD23-, CyclinD1-, Ki-67 5%-15%	Radiotherapy	N	4
18	14/2008	57/F	L/0.5	Conjunctiva	Choroid	CD20+, Bcl2+, CD5-, CD23-, CD43-, Bcl6-, CyclinD1-, Kappa LC+	Radiotherapy	N	6
19		85/M	NA	N	Choroid	CD20+, CD19+, CD5-, Lambda LC+	Radiotherapy	N	3
20	15/2009	68/M	L/HM	Conjunctiva	Choroid	CD20+, CD3±, Lambda LC+, kappa LC-	Radiotherapy	N	60
21	16/2010	80/M	R/0.1	Epibulbar tumor	Iris, ciliary body, choroid	CD3-, CD5-, CD10-, CD20+, CD23-, CD38-, CD43±, CD56-, CD57-, CD79a+, CD138-, Bcl-2+, Bcl-6-, CyclinD1-, IgM-, Kappa LC-, Lambda LC-, Ki-67 < 10%	Enucleation	N	28
22	17/2011	49/M	R/CF	Orbit, sphenoid bone	Choroid	CD20+, CD3-, CD5-, CD10-	Chemotherapy Radiotherapy	N	NA
23	18/2012	62/M	L/0.67	Orbit, optic nerve	Choroid	CD79+, CD20+, BCL2+, CD10+ (focally), CyclinD1-	Radiotherapy	N	6
24	19/2013	73/F	NA/0.625	Conjunctiva, orbit	Choroid	CD20+++, CD5+, Bcl6+, CyclinD1+, Kappa LC+, Lambda LC -, Ki67 5-10%	Radiotherapy	N	6
25		46/M	NA/0.2	N	Choroid	CD20+++, CD79a+++, CD3+	Chemotherapy	N	84
26		63/M	NA/0.16	Conjunctiva	Choroid	CD20+++, CD5+, CD10-, Bcl6-, CyclinD1-, Ki67 50%	Radiotherapy Chemotherapy	N	60
27		54/F	NA/0.625	N	Choroid	CD20+++, CD5+, CD23-, Bcl6-, CyclinD1-, Kappa LC+, Lambda LC+++	Radiotherapy	N	144
28		64/F	NA/0.005	Orbit	Choroid	CD20+++, CD5+, CD10-, CD138+, Bcl6-, CyclinD1-, Kappa LC+, Lambda LC+++, Ki67 5%	Radiotherapy	N	12
29		54/F	NA/0.5	Orbit	Choroid	CD20+, CD5+, CD3+, kappa LC-, Lambda LC-	Radiotherapy Chemotherapy	N	120
30		40/M	NA/0.8	N	Choroid	CD20+, Bcl2-, Kappa LC+	Radiotherapy Chemotherapy	N	156
31		53/F	NA/0.1	Lacrymal gland	Choroid	CD20++, CD3+, CD5+, CD8+, Bcl2+++, Bcl6++, Kappa LC+++, Lambda LC+, Ki67-	Chemotherapy	N	53
32		57/M	NA/0.32	Conjunctiva	Choroid	CD20+++, CD5+, CD23-, Bcl6+, Kappa LC-, Lambda LC-, Ki67 20%	Chemotherapy	N	33
33	20/2013	71/M	L/0.2	Conjunctiva, optic nerve	Choroid	CD20+, CD43+, Bcl2+, CD3-	Chemotherapy	N	12
34	21/2013	71 /F	L/0.5	Conjunctiva	Iris, choroid	NA	Radiotherapy	N	44
35		71/M	R/0.05	Conjunctiva	Iris, choroid	NA	Radiotherapy	N	36
36		75/M	R/0.5	Conjunctiva, orbit	Iris, ciliary body, choroid	NA	Radiotherapy	Unknown	0
37-52	22/2014	NA	NA	NA	Choroid	NA	NA	NA	NA
53-60	23/2014	NA	NA	NA	NA	NA	NA	NA	NA
61	Present case	38/M	R/0.1	Orbit, optic nerve	Iris, ciliary body, choroid, trabecular meshwork	CD5+, CD10-, CD20+, CD23+, CD38+, CD79a+, Bcl-2+, Bcl-6-, CyclinD1-, Kappa LC+, Lambda LC+, Ki-67 5%-10%	Enucleation	N	18

BCVA, best corrected visual acuity; CF, counting figure; F, female; HM, hand motion; L, left; LP, light perception; M, male; N, no; NA, not available; NLP, no light perception; R, right; Y, yes.

Clinical diagnosis of early EMZL may be difficult. Uveal lymphoma can masquerade as amelanotic uveal melanoma, uveal metastases, uveal effusion syndrome, posterior scleritis, and various uveitities. This presents a significant diagnostic challenge [25-27]. Delays in the diagnosis of uveal lymphoma are common for its insidious and nonspecific onset [16]. Loriaut et al. reported a series of choroidal and adnexal extranodal marginal zone B-cell lymphoma. In their 9 cases, the mean time to diagnosis was 12 months after onset and the longest period for diagnosis delay was 24 months [19]. Typical presenting symptoms such as recurrent episodes of blurred vision, painless visual loss and metamorphopsia, may result from macular serous retinal detachment [10,12]. The characteristic findings include clear vitreous, choroidal thickening and no involvement of central nervous system. Imaging techniques, including ultrasonography and orbital MRI, are valuable to detect extraocular involvement [28]. Histopathologic study with immunohistochemical stains remains mandatory to make an accurate diagnosis and classification [12]. Biopsy of the episcleral tumour nodule or an intraocular biopsy is commonly required to determine the nature of the uveal mass [29]. However, it is difficult to obtain a tissue sample. In some inconclusive or late-stage cases, it may be necessary to perform enucleation for the definitive diagnosis [13,30]. In the present case, absence of any evidence of systemic disease at presentation and during 2 years of follow-up, could have facilitated the diagnosis of primary EMZL of the uvea.

The exact incidence of primary iris and ciliary body lymphoma remains unknown because only a small number of cases have been reported. This may be due to the evidence that most iris and ciliary body infiltrations have been confirmed only after enucleation or on postmortem histopathology [31]. There were only two reports with diffuse uveal involvement in our literature review. However, no infiltration of trabecular meshwork was mentioned [16,21]. In this case we report diffuse uveal involvement (choroid, ciliary body and iris) with focal trabecular meshwork infiltration, which is the first report to the best of our knowledge. However, no secondary glaucoma was found in this case. The mechanism was uncertain and infiltration in early stage may be one of the possible explanations. Our case also masqueraded as posterior scleritis or choroidal hemangioma, presenting diffuse thickened choroid with optic nerve and orbit involvement.

To date, there are no consensus to the optimal treatment for uveal lymphoma due to the small number of such patients and short duration of follow-up. Some authors preferred low-dose external radiotherapy for uveal lymphoma on the basis of satisfactory disease control and few side effects [12]. Although conventional external radiotherapy remains the most frequently used modality, exclusive chemotherapy based on anti-CD20 regimen increasingly becomes one of the treatment choices [19,32].

## Conclusions

In summary, uveal EMZL may present with various features and we report a rare case with diffuse involvement of the uveal and focal trabecular meshwork infiltration. The diagnosis of uveal EMZL is challenging and commonly delayed due to its insidious and nonspecific onset. Early clinical recognition, tissue biopsy, and expert cytologic assessment are critical to achieve early diagnosis and better clinical outcomes. Despite the rarity, uveal EMZL should be included in the differential diagnosis of uveal inflammation or tumors, particularly in patients with persistent signs and symptoms. Non-resolving or recurrent masquerade syndromes not responding to standard treatment should lead to prompt biopsy [33].

## Consent

Written informed consent was obtained from the patient for publication of this Case Report and any accompanying images. A copy of the written consent is available for review by the Editor of this journal.

## Abbreviations

EMZL: Extranodal marginal zone lymphoma
RPE: Retinal pigment epithelium
MALT: Mucosa-associated lymphoid tissue
